# Supplementary material for: Abnormal developmental trajectory and vulnerability to cardiac arrhythmias in tetralogy of Fallot with DiGeorge syndrome
Source: Commun Biol. 2023 Sep 22;6:969. doi: 10.1038/s42003-023-05344-6 (PMC10516936; doi:10.1038/s42003-023-05344-6)
Supplement: Supplementary file 2 — Supplemental Information [file 42003_2023_5344_MOESM2_ESM.pdf]

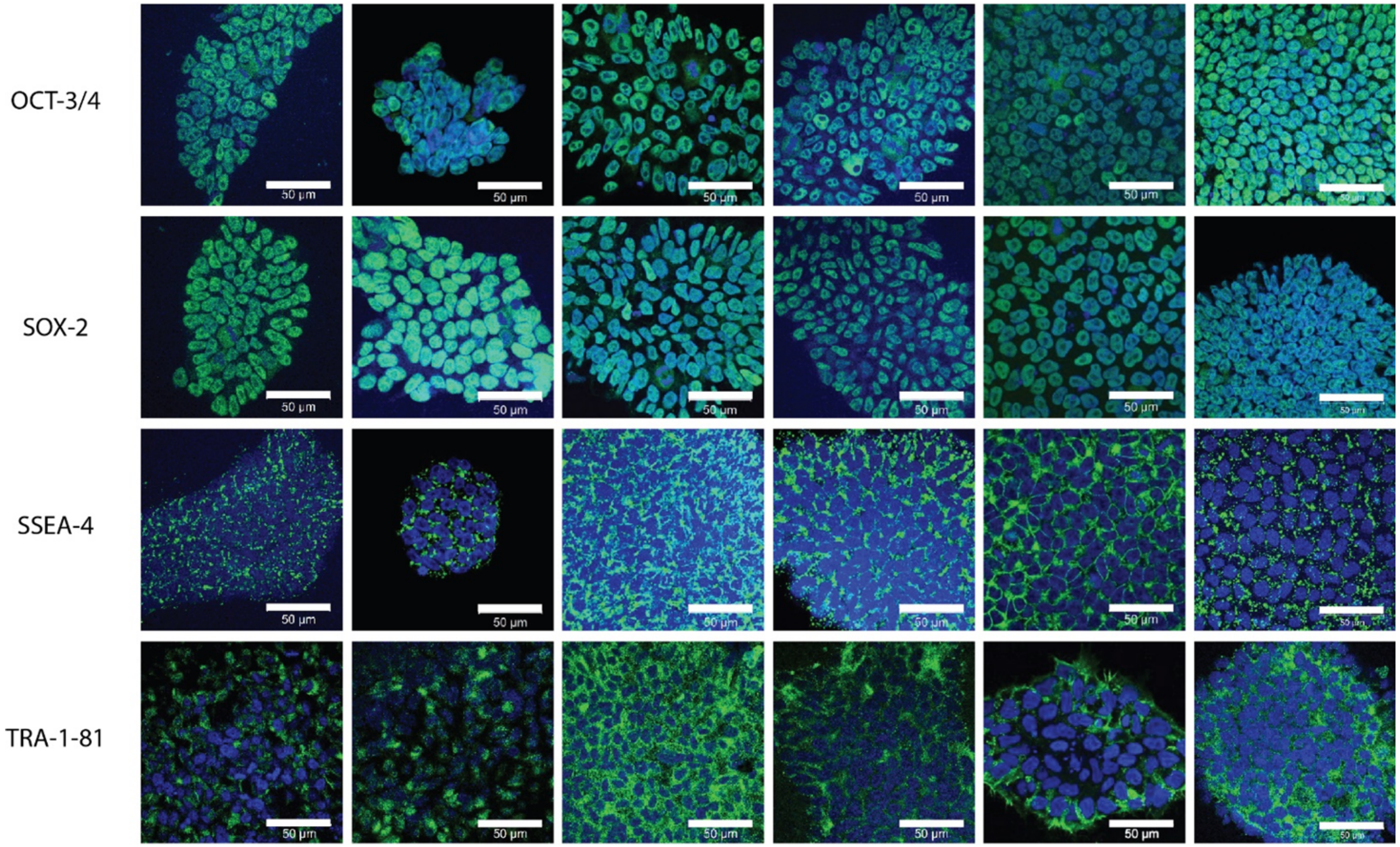


Supplemental Figure 1 Immunohistochemistry of pluripotency markers in hiPSC

Representative immunofluorescences of four different pluripotency markers in hiPSC, OCT-3/4 (green, 1st row); SOX-2 (green, 2nd row); SSEA-4 (green, 3rd row), TRA-1-81(green, 4th row) and DAPI (blue). Scale bars, 50um.

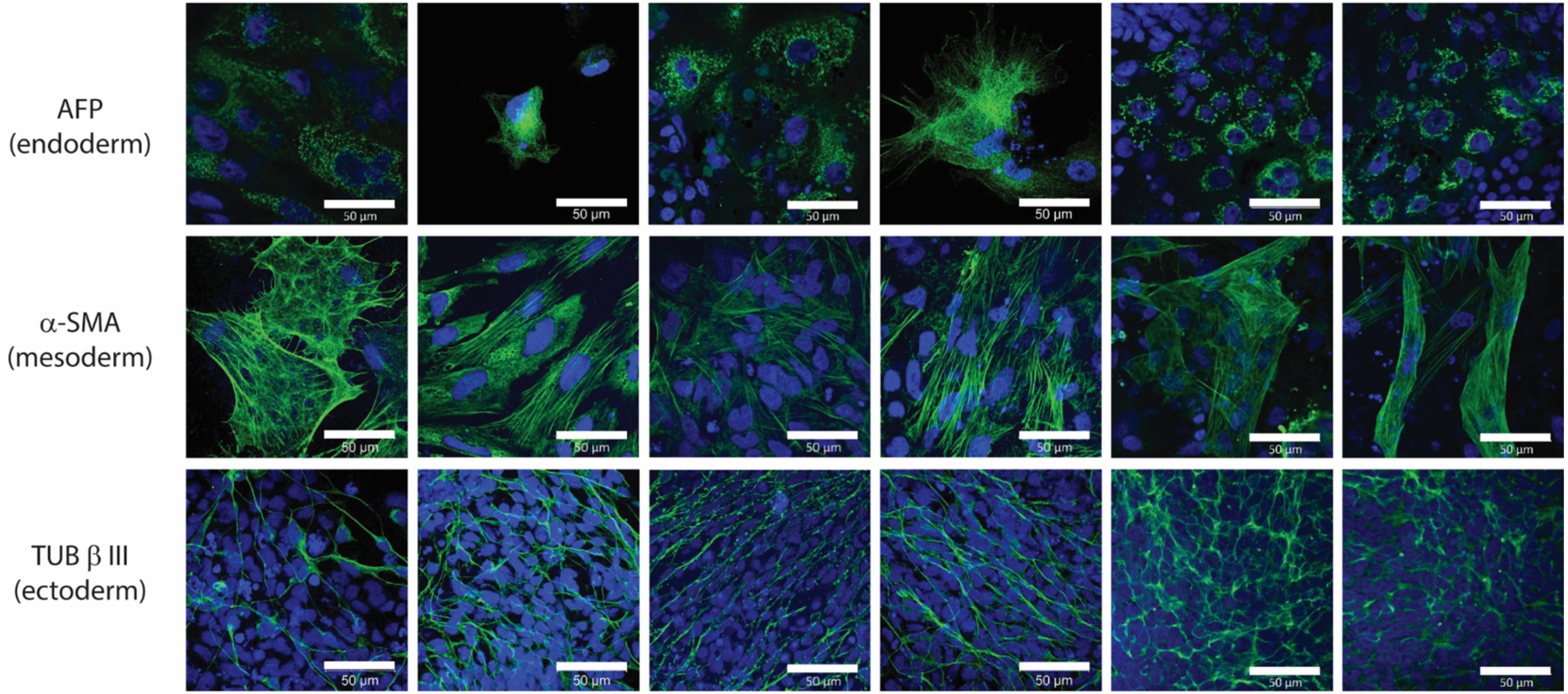


Supplemental Figure 2 Immunohistochemistry of germ layer markers in hiPSC

Representative immunofluorescences of three different germ layer markers in spontaneous embryoid body formation from iPSC, AFP (green, top panel); α-SMA (green, middle panel); TUB β III (green, lower panel) and DAPI (blue). Scale bars, 50um.

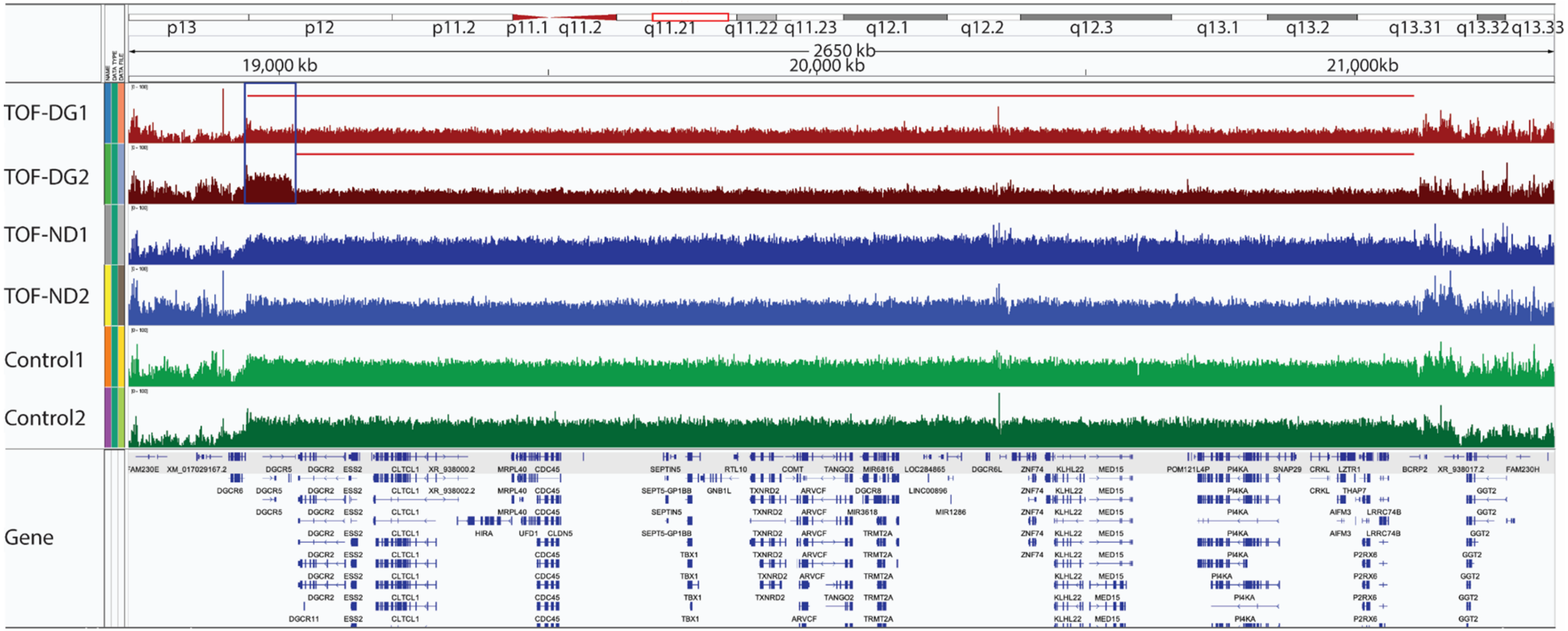


Supplemental Figure 3 Whole-genome sequencing coverage of hiPSC lines in chromosome 22

Within the region of chromosome 22q11.2, near 20Mb, readings mapped in both TOF-DG-hiPSC lines were half of those mapped in TOF-ND/control-hiPSC lines. The regions of haploinsufficiency were indicated by the arrows. The haploinsufficiency was cross-validated with different methods, including Runs of homozygosity, Lumpy and GRIDSS. Mb, million base pairs.

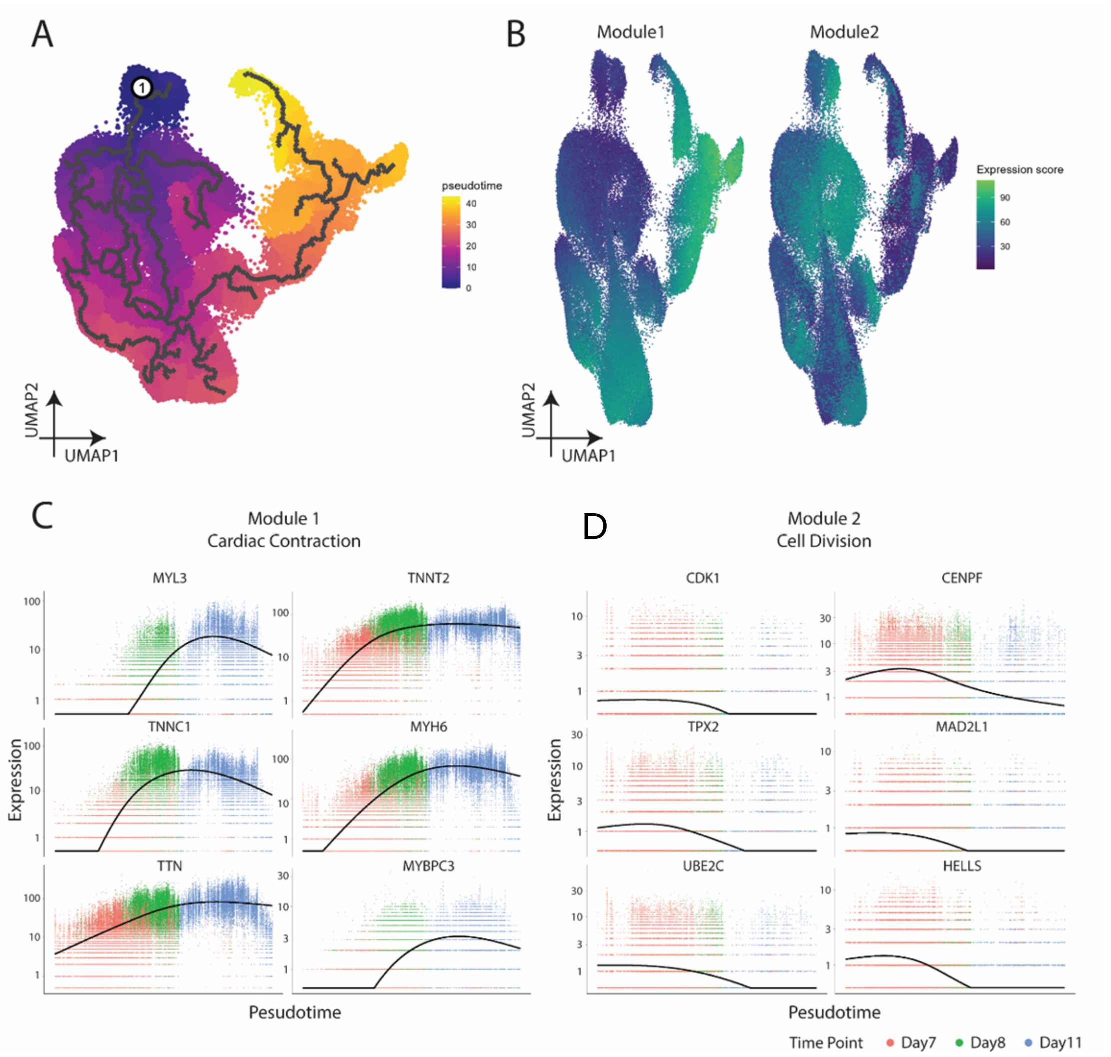


Supplemental Figure 4 Trajectory analysis hiPSC-CPs and immature hiPSC-CMs

(A) Pseudotime analysis of the hiPSC-CPs and immature hiPSC-CMs. Root node was denoted with ®. Trajectory ended at Day11-immature hiPSC-CMs.

(B) Gene modules serve as the function of the pesudotime analysis and trajectory inference of the hiPSC-CPs and immature hiPSC-CMs.

(C) (D) Gene expression dynamics (corresponding to the GO terms enriched in module 1 & 2) along the trajectory. X-axis: Expression level; Y-axis: pseudotime.

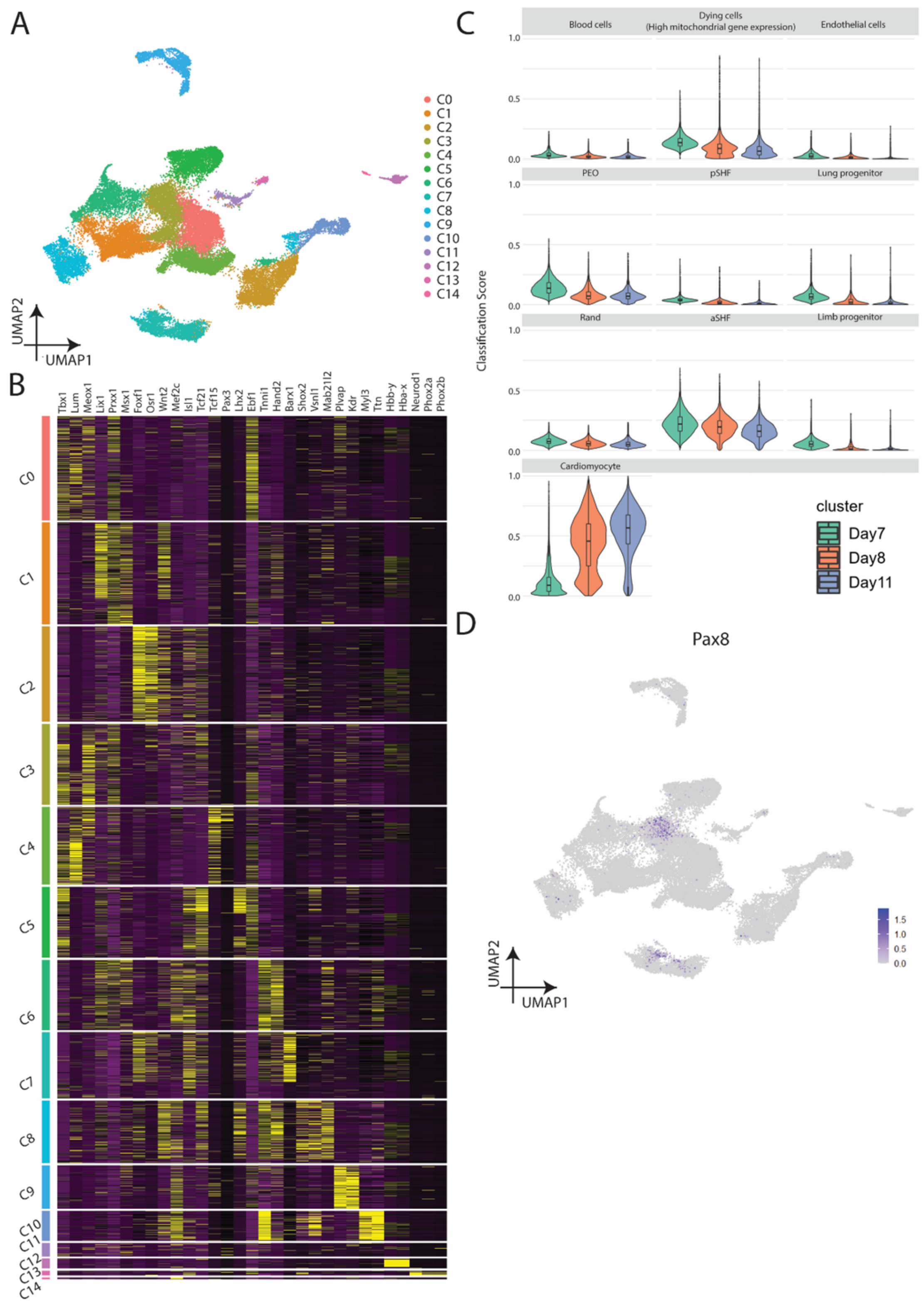


Supplemental Figure5 Cross-species comparison between hiPSC-CPs/immature CMs with *Tbx1*-cko mouse dataset

(A) UMAP plot of *Tbx1*-cko mouse dataset. C0: Mesenchyme; C1: Limb progenitors; C2: pSHF; C3: MLP; C4: Paraxial mesoderm; C5: Branchiomeric muscle progenitor; C6: aSHF; C7: Lung progenitor; C8: PEO; C9: Endothelial cells; C10: cardiomyocytes; C11: Dying cells with high mitochondrial gene expression; C12: blood cells with high

haemoglobin expression; C13: Neural progenitor; C14: blood cells with low haemoglobin expression.

(B) Heatmap presentations of the marker genes expressed in each cluster.

(C) Classification score of hiPSC-CPs/immature CMs to *Tbx1*-cko mouse progenitors. Classifications with score >0.2 in any of the samples (D7/8 hiPSC-CPs and D11 immature CMs) were shown as violin plot.

(D) UMAP plot of ectopic *Pax8* expression in *Tbx1*-cko mouse MLP and lung progenitors.

| | Sex | Date of surgical repair of TOF | The need for subsequent pulmonary valve replacement | Arrhythmia | Association |
|---|---|---|---|---|---|
| **TOF-DG1** | Male | 1991 | + | Nil | hypoparathyroidism, hypocalcaemia |
| **TOF-DG2** | Female | 1997 | - | Runs of wide complex tachycardia, ventricular premature beats | - |
| **TOF-ND1** | Male | 1991 | + | Atrial and ventricular premature beats | - |
| **TOF-ND2** | Female | 1980 | + | Supraventricular tachycardia | - |
| **Control1** | Male | - | - | - | - |
| **Control2** | Female | - | - | - | - |

Supplemental Table 1. Clinical phenotypes of the subjects for hiPSC generation

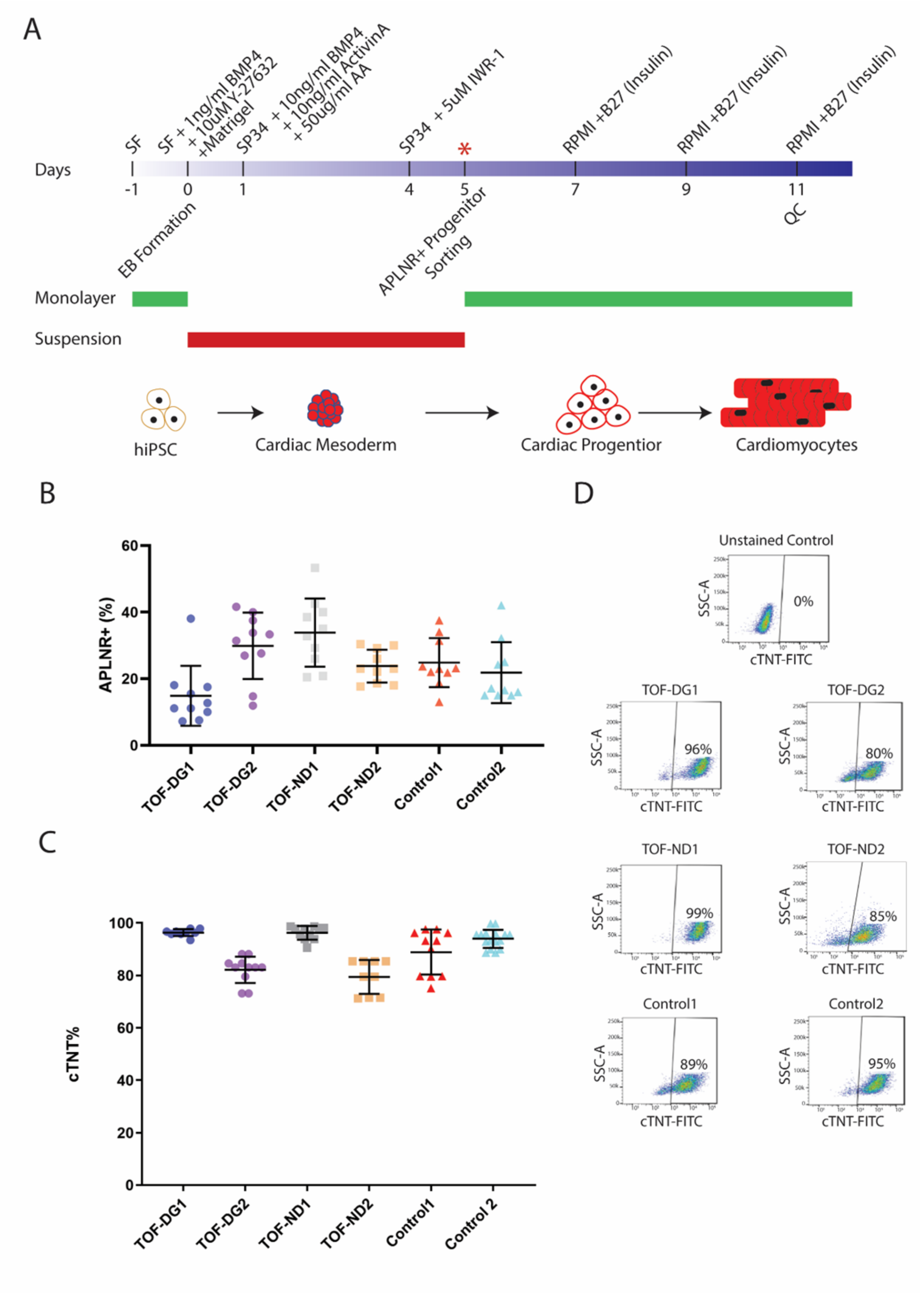


Supplemental Figure 6 Cardiac Differentiation.

(A) Schematics of APLNR$^{+}$ sorting *in vitro* cardiac differentiation

(B) Percentage of APLNR$^{+}$ progenitors in 10 batches of cardiac differentiation

(C) Percentage of cardiomyocyte (cTNT$^{+}$) used for CAS construction

(D) Representative flow cytometry analysis of cardiomyocyte (cTNT/FITC-A)

Data were presented in Mean $\pm$ SD for Fig. S6B&S6C.
