# Supplementary material for: Abnormal developmental trajectory and vulnerability to cardiac arrhythmias in tetralogy of Fallot with DiGeorge syndrome
Source: Commun Biol. 2023 Sep 22;6:969. doi: 10.1038/s42003-023-05344-6 (PMC10516936; doi:10.1038/s42003-023-05344-6)
Supplement: Supplementary file 3 — Description of Additional Supplementary Files [file 42003_2023_5344_MOESM3_ESM.pdf]

## Description of Additional Supplementary Files

**File name:** Supplementary Data 1
**Description:** An excel file supporting the bioinformatic analysis on the single cell RNA sequencing in the paper.

**File name:** Supplementary Data 2
**Description:** An example of flow cytometry analysis, including raw data, analysis files, read_me.txt file describing the content.

**File name:** Supplementary Data 3
**Description:** Source data and summary of CAS measurements. Data is separated into folders named after batch, cell line, replicate (A1,A2,etc..) and effective refractory period (if arrhythmia occurred, it was named with arrhythmia/arrhythmic instead). Action potential (1A-wave.csv) and calcium transient (1B-wave.csv) at 1Hz were included.
