# Supplementary figures and images for: Abnormal developmental trajectory and vulnerability to cardiac arrhythmias in tetralogy of Fallot with DiGeorge syndrome
Source: Commun Biol. 2023 Sep 22;6:969. doi: 10.1038/s42003-023-05344-6 (PMC10516936; doi:10.1038/s42003-023-05344-6)

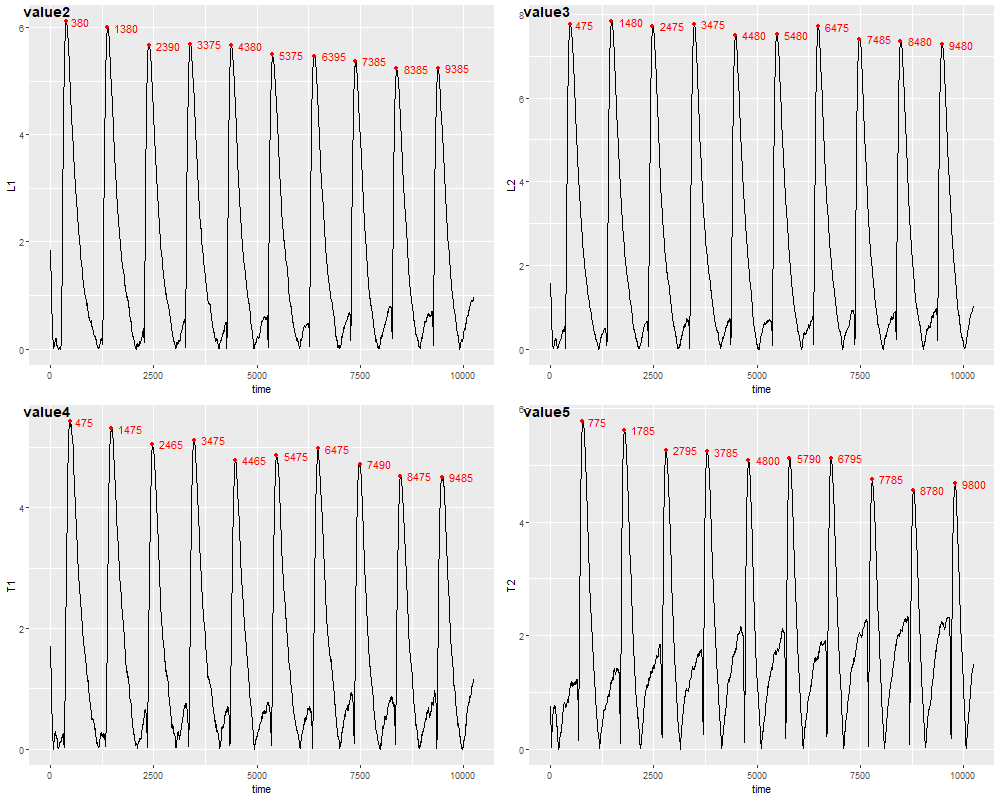

Supplement: Supplementary file 6 — Supplementary Data 3 [file 42003_2023_5344_MOESM6_ESM.zip › Supplementary Data3_CAS Measurement/Batch60/TOF-DG2_A1_arrythmia/Ca.png]

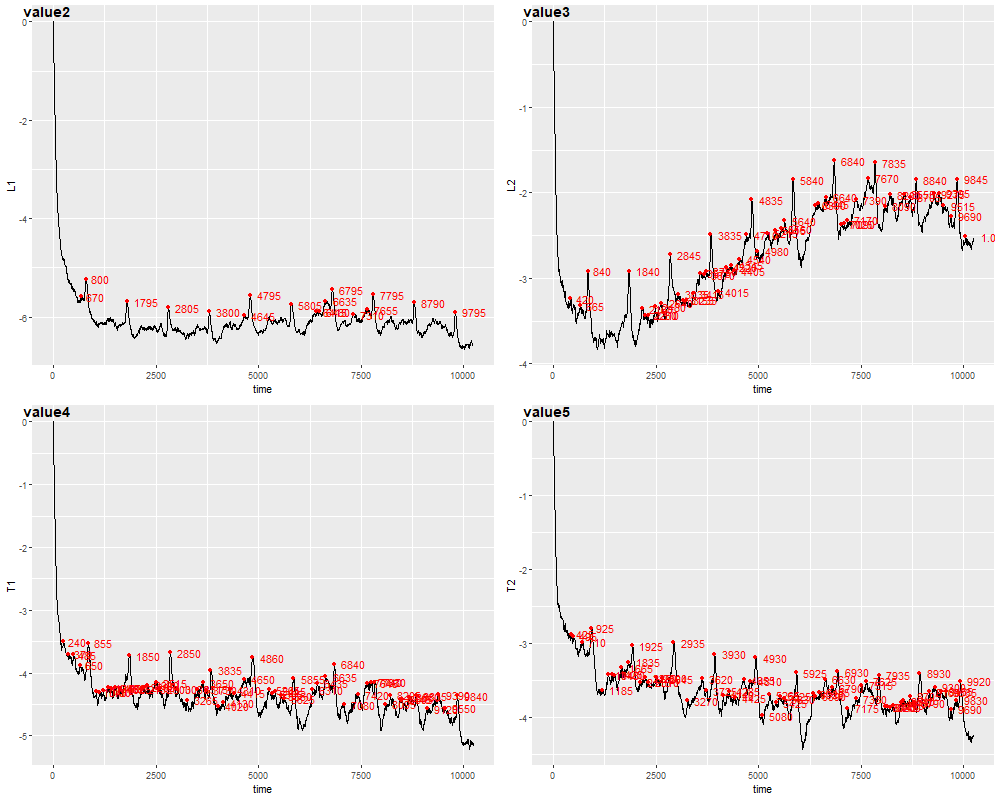

Supplement: Supplementary file 6 — Supplementary Data 3 [file 42003_2023_5344_MOESM6_ESM.zip › Supplementary Data3_CAS Measurement/Batch61/TOF-DG2_A1_arrythmia_4815/AP.png]

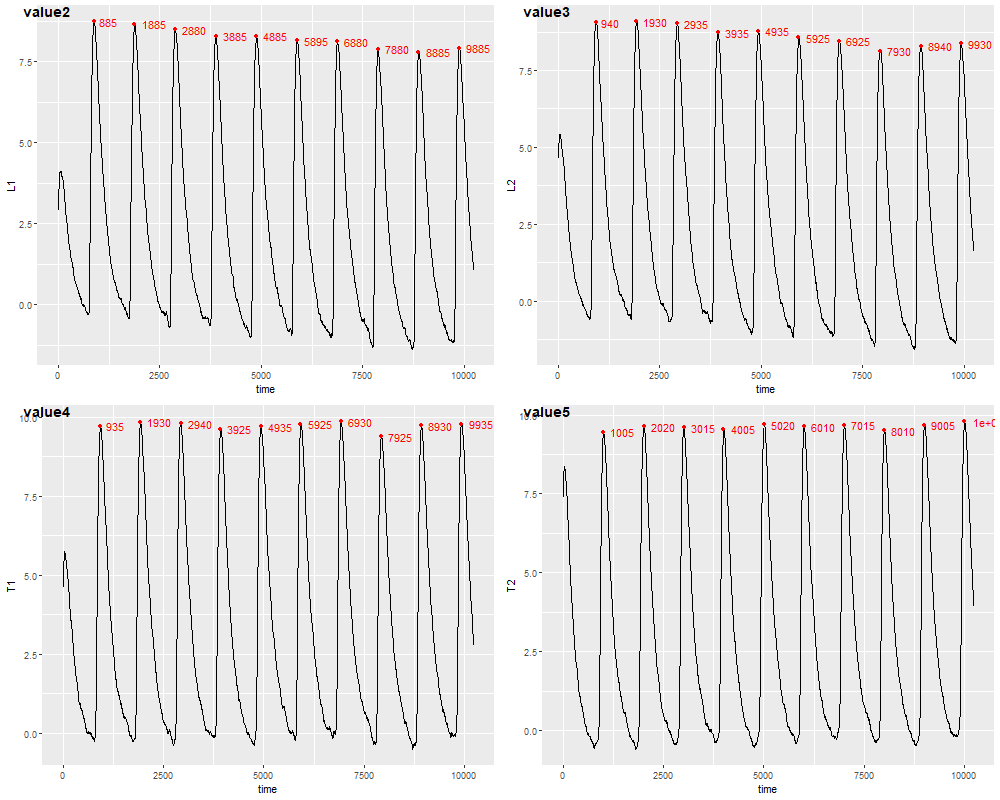

Supplement: Supplementary file 6 — Supplementary Data 3 [file 42003_2023_5344_MOESM6_ESM.zip › Supplementary Data3_CAS Measurement/Batch61/TOF-DG2_A1_arrythmia_4815/Ca.png]

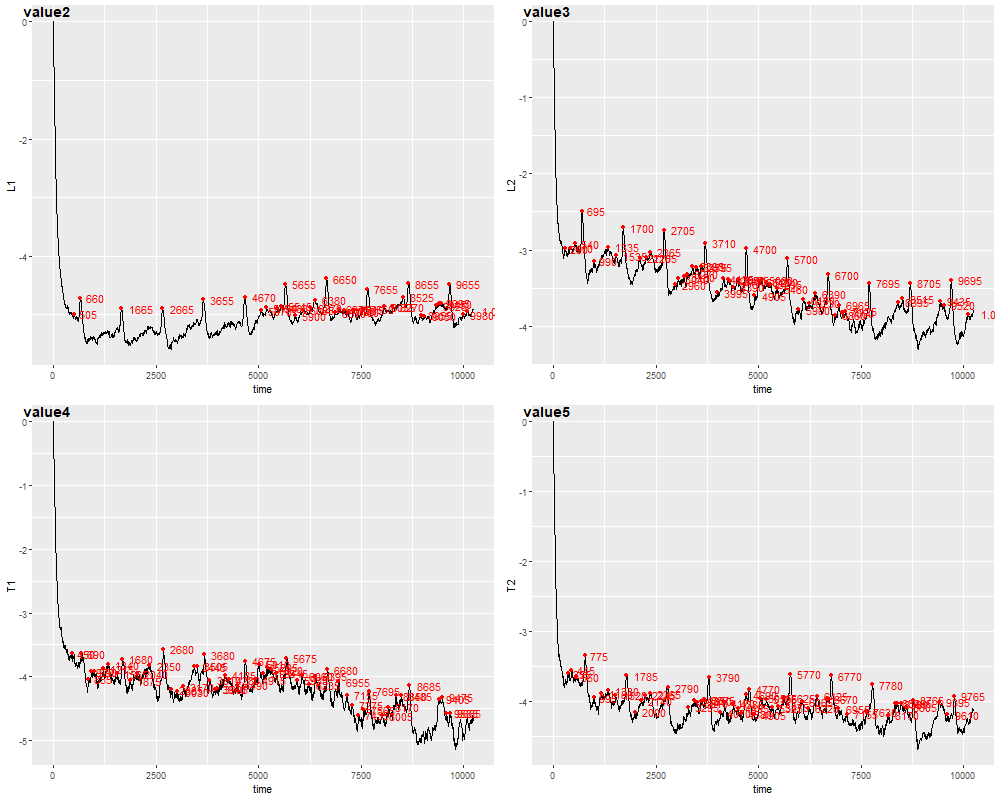

Supplement: Supplementary file 6 — Supplementary Data 3 [file 42003_2023_5344_MOESM6_ESM.zip › Supplementary Data3_CAS Measurement/Batch61/TOF-DG2_A2_4810/AP.png]

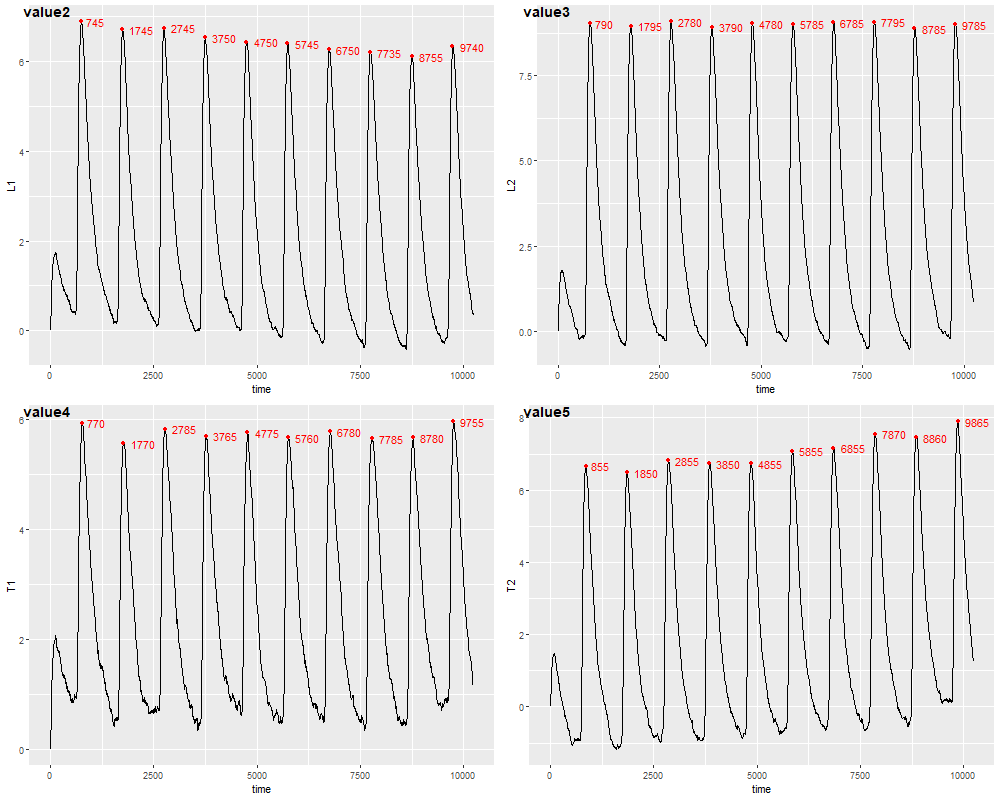

Supplement: Supplementary file 6 — Supplementary Data 3 [file 42003_2023_5344_MOESM6_ESM.zip › Supplementary Data3_CAS Measurement/Batch61/TOF-DG2_A2_4810/Ca.png]

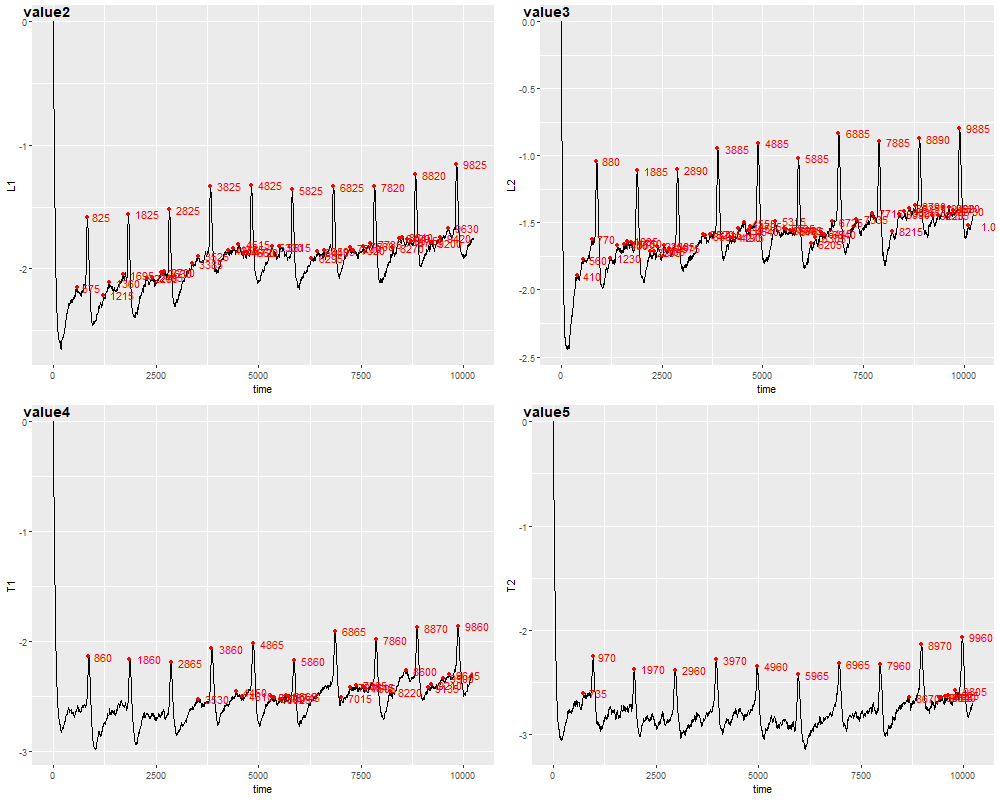

Supplement: Supplementary file 6 — Supplementary Data 3 [file 42003_2023_5344_MOESM6_ESM.zip › Supplementary Data3_CAS Measurement/Batch61/TOF-DG2_A3_arrythmia_4900/AP.png]

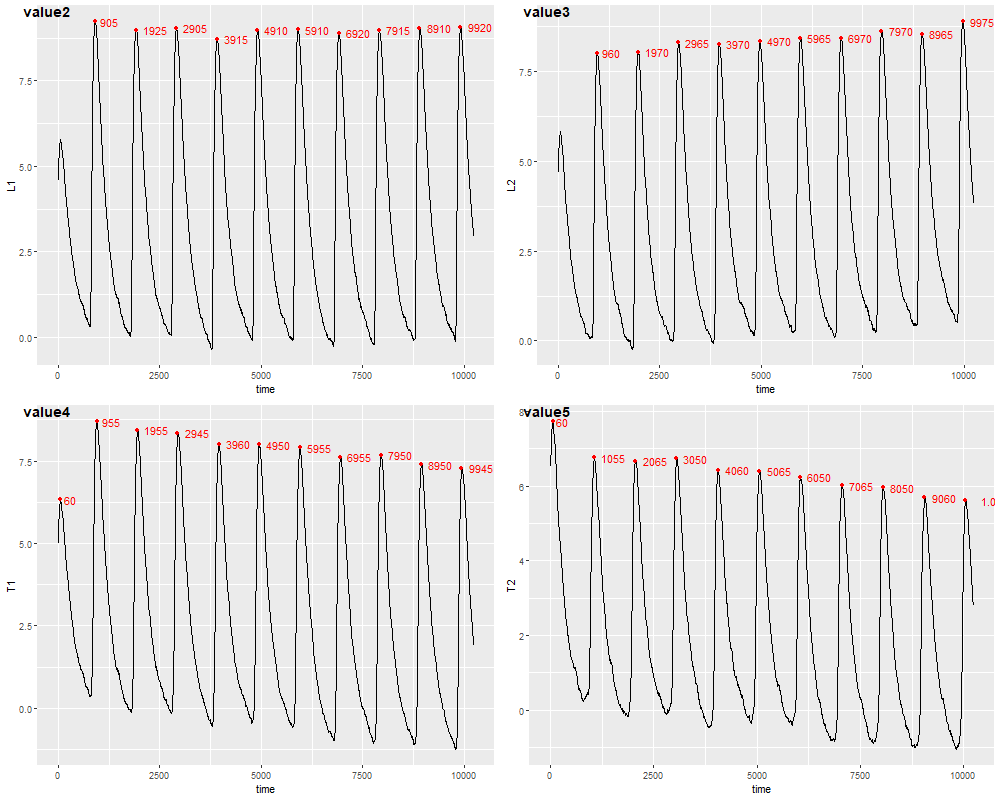

Supplement: Supplementary file 6 — Supplementary Data 3 [file 42003_2023_5344_MOESM6_ESM.zip › Supplementary Data3_CAS Measurement/Batch61/TOF-DG2_A3_arrythmia_4900/Ca.png]

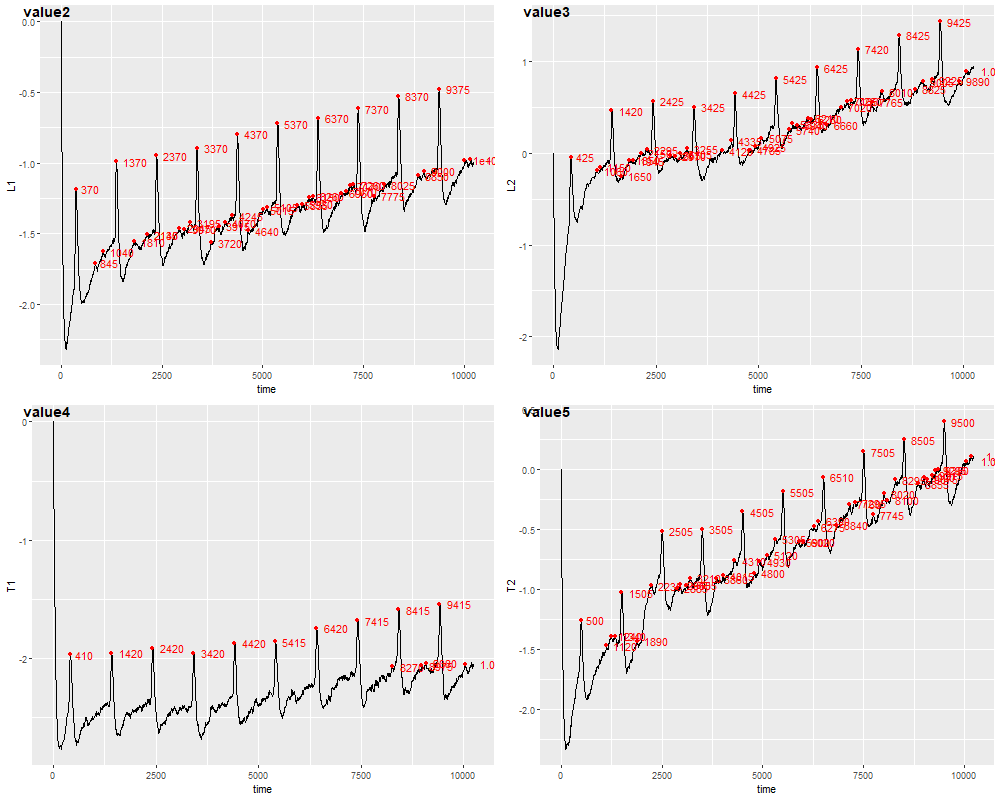

Supplement: Supplementary file 6 — Supplementary Data 3 [file 42003_2023_5344_MOESM6_ESM.zip › Supplementary Data3_CAS Measurement/Batch61/TOF-DG2_A4_arrythmia_4850/AP.png]

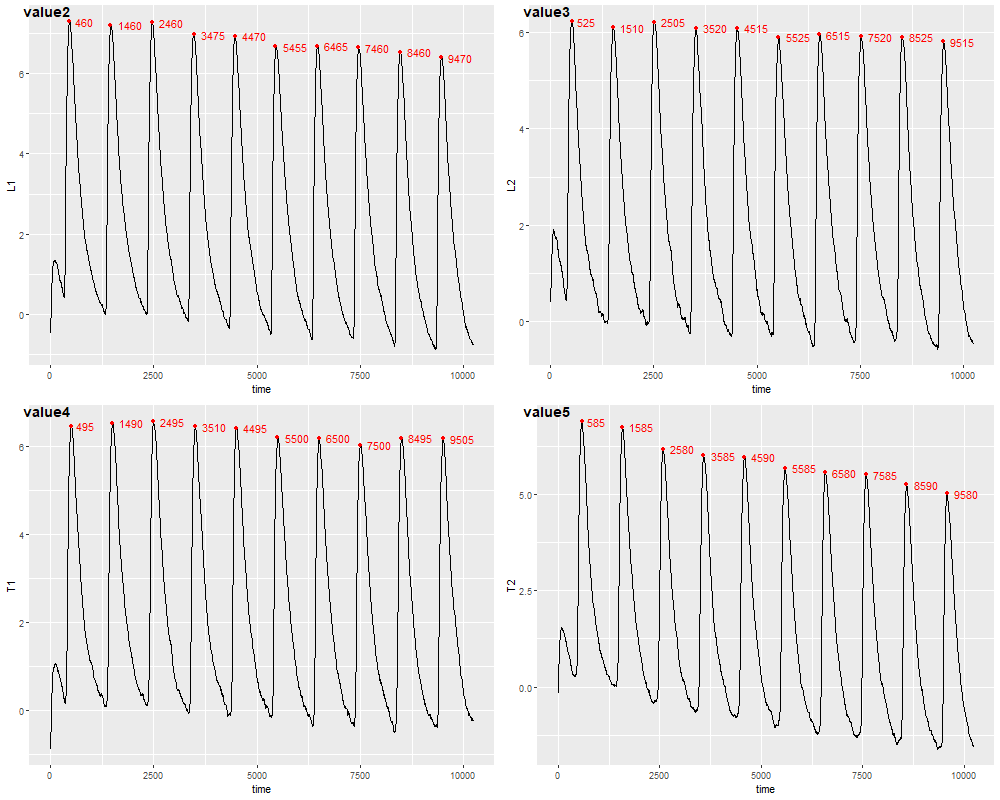

Supplement: Supplementary file 6 — Supplementary Data 3 [file 42003_2023_5344_MOESM6_ESM.zip › Supplementary Data3_CAS Measurement/Batch61/TOF-DG2_A4_arrythmia_4850/Ca.png]

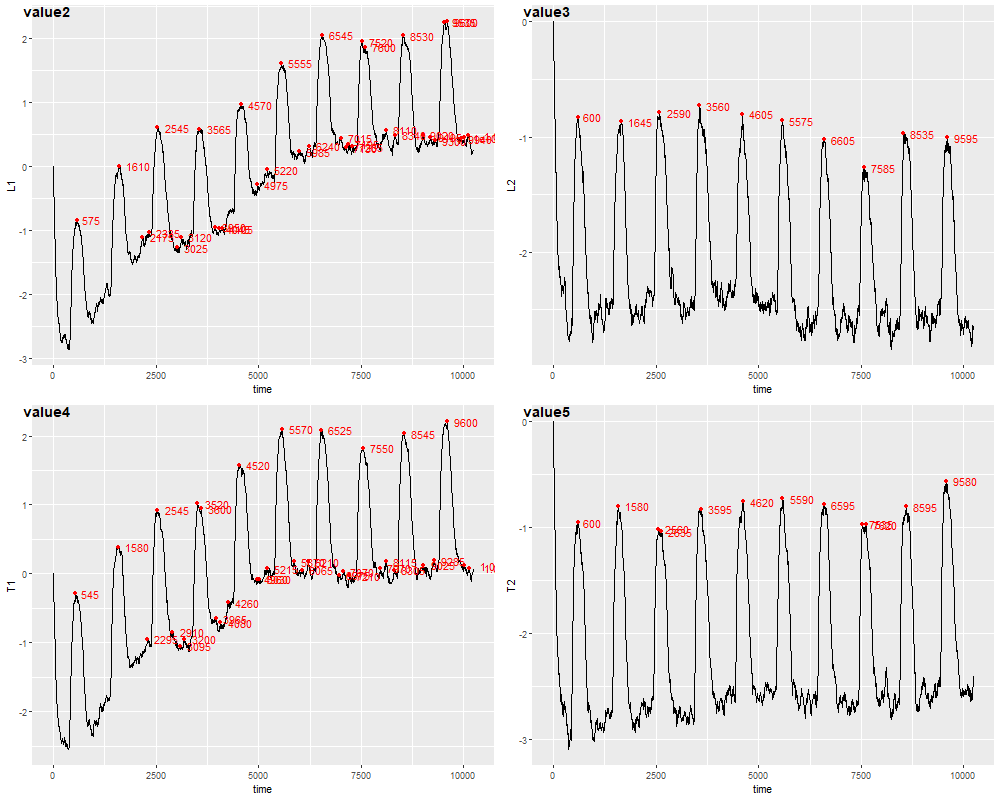

Supplement: Supplementary file 6 — Supplementary Data 3 [file 42003_2023_5344_MOESM6_ESM.zip › Supplementary Data3_CAS Measurement/Batch61/TOF-ND2_A1_5050/AP.png]

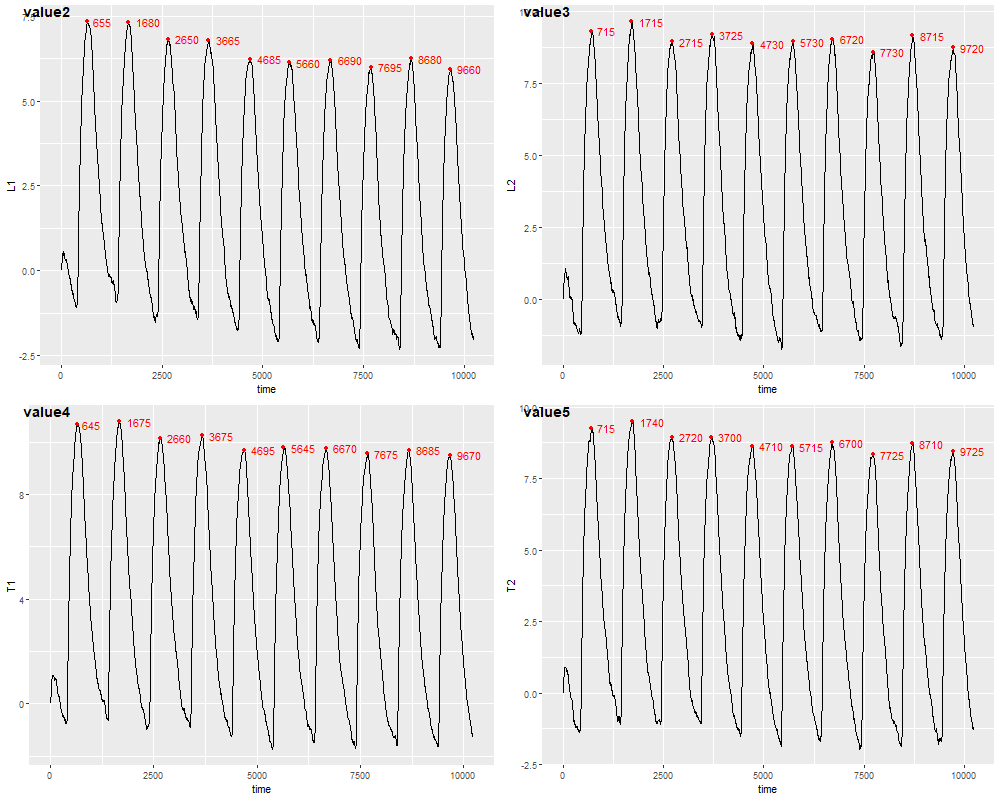

Supplement: Supplementary file 6 — Supplementary Data 3 [file 42003_2023_5344_MOESM6_ESM.zip › Supplementary Data3_CAS Measurement/Batch61/TOF-ND2_A1_5050/Ca.png]

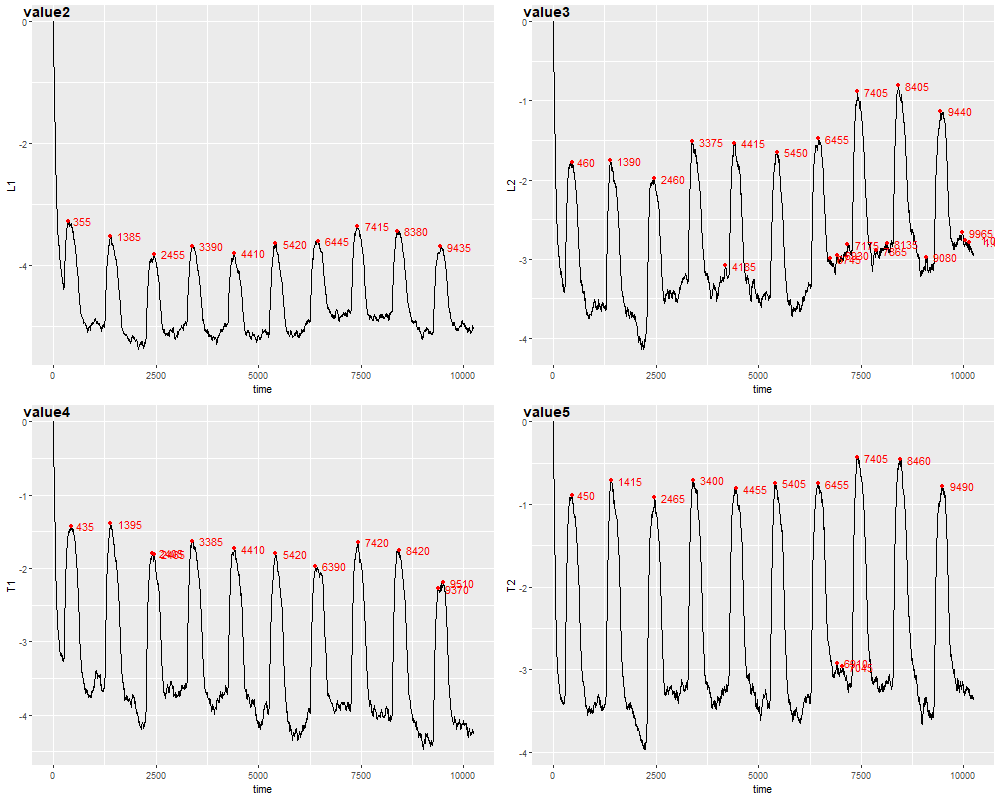

Supplement: Supplementary file 6 — Supplementary Data 3 [file 42003_2023_5344_MOESM6_ESM.zip › Supplementary Data3_CAS Measurement/Batch61/TOF-ND2_A2_5035/AP.png]

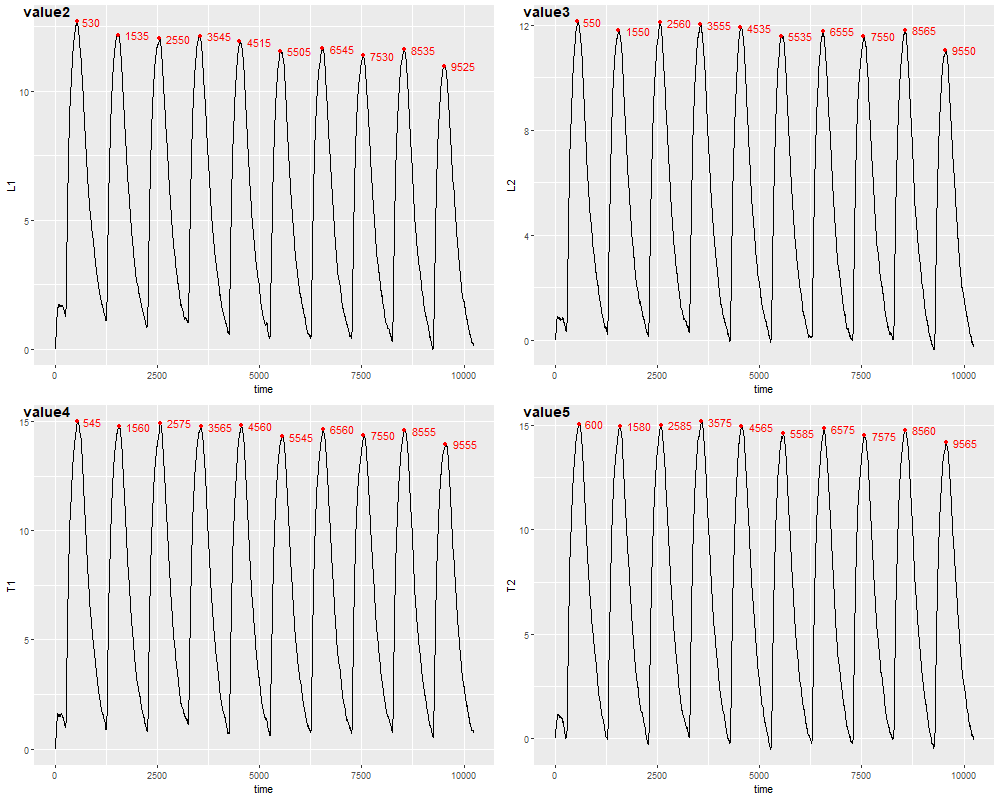

Supplement: Supplementary file 6 — Supplementary Data 3 [file 42003_2023_5344_MOESM6_ESM.zip › Supplementary Data3_CAS Measurement/Batch61/TOF-ND2_A2_5035/Ca.png]

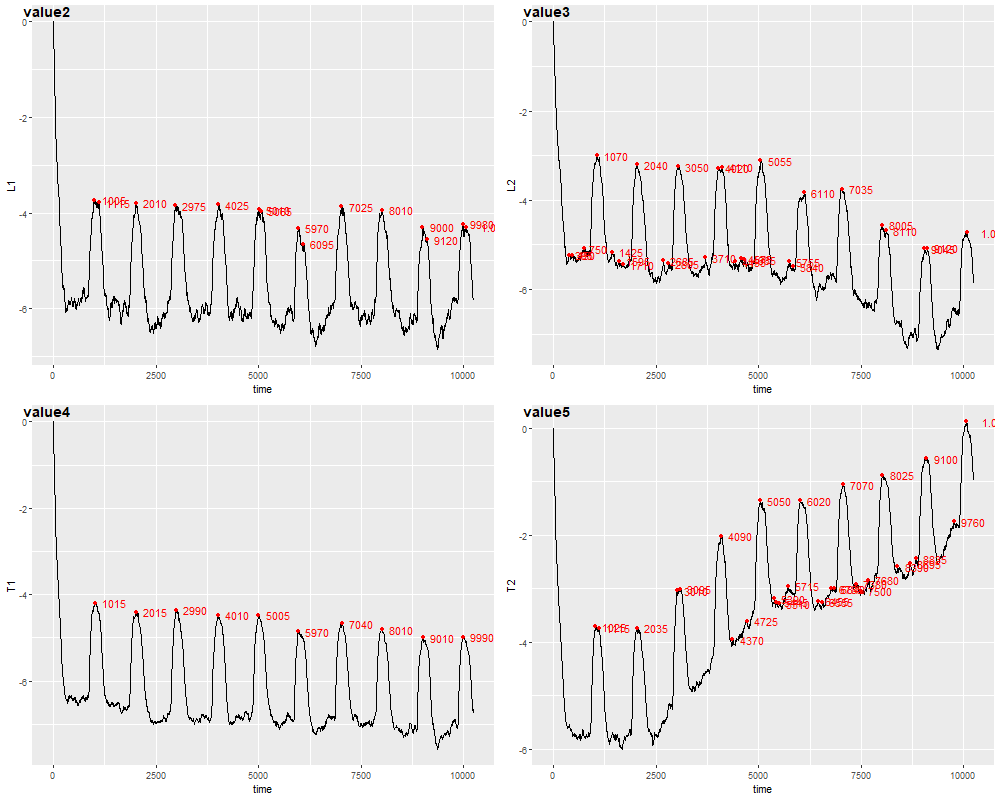

Supplement: Supplementary file 6 — Supplementary Data 3 [file 42003_2023_5344_MOESM6_ESM.zip › Supplementary Data3_CAS Measurement/Batch61/TOF-ND2_A3_5035/AP.png]

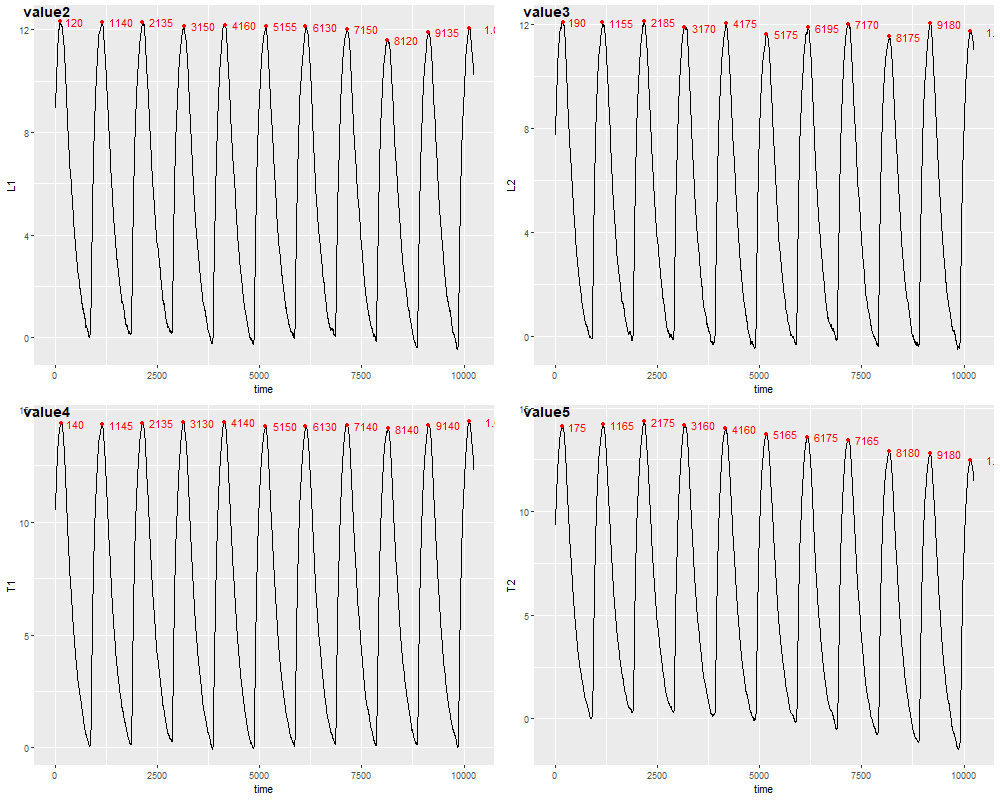

Supplement: Supplementary file 6 — Supplementary Data 3 [file 42003_2023_5344_MOESM6_ESM.zip › Supplementary Data3_CAS Measurement/Batch61/TOF-ND2_A3_5035/Ca.png]

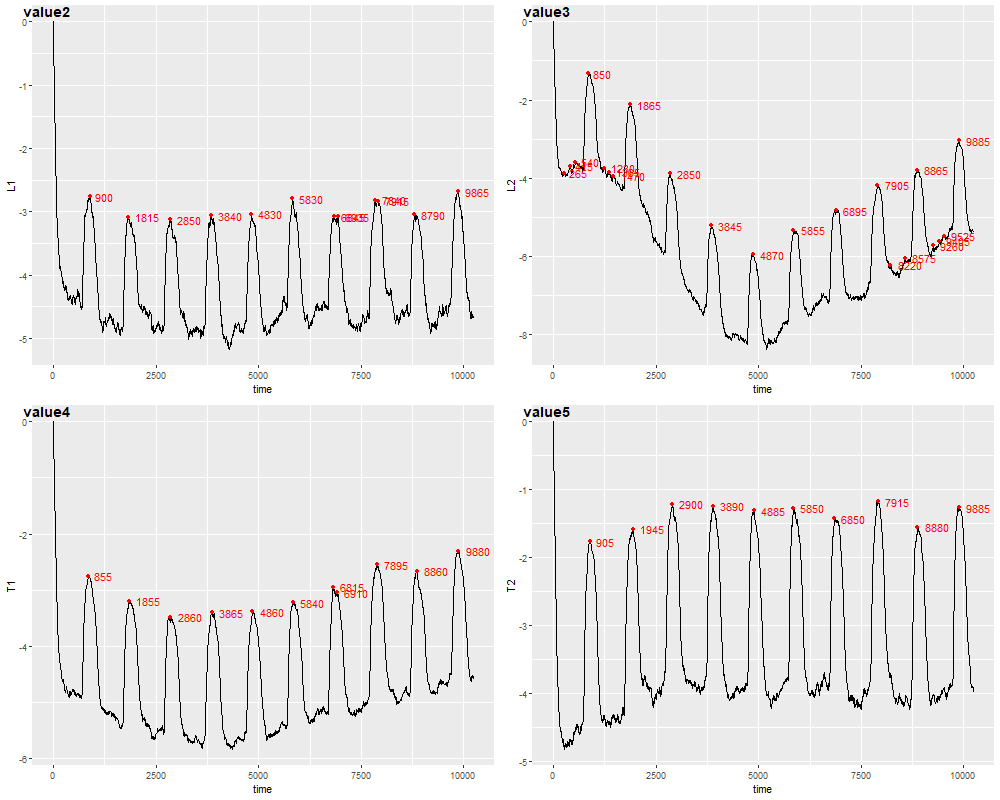

Supplement: Supplementary file 6 — Supplementary Data 3 [file 42003_2023_5344_MOESM6_ESM.zip › Supplementary Data3_CAS Measurement/Batch61/TOF-ND2_A4_5050/AP.png]

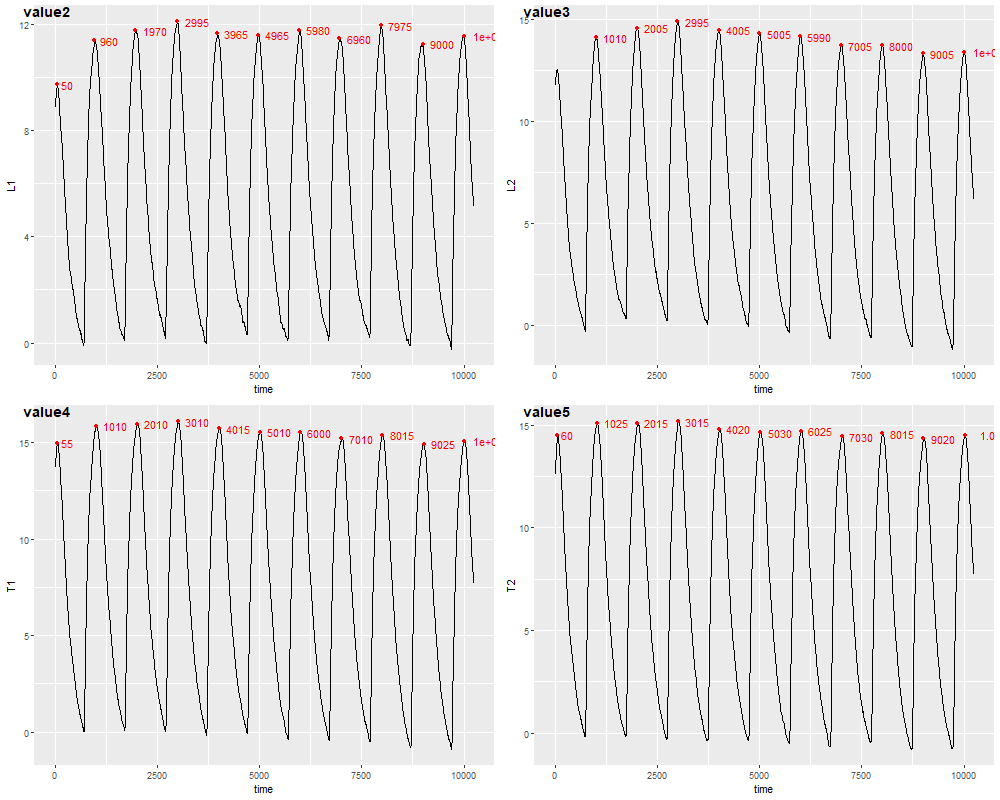

Supplement: Supplementary file 6 — Supplementary Data 3 [file 42003_2023_5344_MOESM6_ESM.zip › Supplementary Data3_CAS Measurement/Batch61/TOF-ND2_A4_5050/Ca.png]
