# Supplementary material for: Abnormal developmental trajectory and vulnerability to cardiac arrhythmias in tetralogy of Fallot with DiGeorge syndrome
Source: Commun Biol. 2023 Sep 22;6:969. doi: 10.1038/s42003-023-05344-6 (PMC10516936; doi:10.1038/s42003-023-05344-6)
Supplement: Supplementary file 7 — Reporting Summary [file 42003_2023_5344_MOESM7_ESM.pdf]

nature portfolio

Corresponding author(s): DBPR, COMMSBIO-22-1746C

Last updated by author(s): Aug 9, 2023

Reporting Summary

Nature Portfolio wishes to improve the reproducibility of the work that we publish. This form provides structure for consistency and transparency in reporting. For further information on Nature Portfolio policies, see our Editorial Policies and the Editorial Policy Checklist.

Statistics

For all statistical analyses, confirm that the following items are present in the figure legend, table legend, main text, or Methods section.

n/a	Confirmed	
☐	☒	The exact sample size (*n*) for each experimental group/condition, given as a discrete number and unit of measurement
☐	☒	A statement on whether measurements were taken from distinct samples or whether the same sample was measured repeatedly
☐	☒	The statistical test(s) used AND whether they are one- or two-sided<br>*Only common tests should be described solely by name; describe more complex techniques in the Methods section.*
☒	☐	A description of all covariates tested
☐	☒	A description of any assumptions or corrections, such as tests of normality and adjustment for multiple comparisons
☐	☒	A full description of the statistical parameters including central tendency (e.g. means) or other basic estimates (e.g. regression coefficient) AND variation (e.g. standard deviation) or associated estimates of uncertainty (e.g. confidence intervals)
☐	☒	For null hypothesis testing, the test statistic (e.g. *F*, *t*, *r*) with confidence intervals, effect sizes, degrees of freedom and *P* value noted<br>*Give P values as exact values whenever suitable.*
☒	☐	For Bayesian analysis, information on the choice of priors and Markov chain Monte Carlo settings
☒	☐	For hierarchical and complex designs, identification of the appropriate level for tests and full reporting of outcomes
☒	☐	Estimates of effect sizes (e.g. Cohen's *d*, Pearson's *r*), indicating how they were calculated

Our web collection on statistics for biologists contains articles on many of the points above.

Software and code

Policy information about availability of computer code

Data collection	No software was used.

Data analysis	MultiQC<br>Trimmomatic (version 0.39)<br>bwa mem<br>SAMtools<br>GATK (version 4.2.0.0)<br>GATK Best Practices<br>VCFtools (version 0.1.16)<br>ANNOVAR (2020-06-07)<br>ClinVar (20210501)<br>lumpy (version 0.2.13)<br>GRIDSS (version 2.13.0)<br>Integrative Genomics Viewer (IGV)<br>Bcftools/Roh<br>Monocle3<br>pySCENIC<br>Harmony<br>SingleCellNet<br>BV Ana imaging software

For manuscripts utilizing custom algorithms or software that are central to the research but not yet described in published literature, software must be made available to editors and reviewers. We strongly encourage code deposition in a community repository (e.g. GitHub). See the Nature Portfolio guidelines for submitting code & software for further information.

Data

Policy information about availability of data

All manuscripts must include a data availability statement. This statement should provide the following information, where applicable:

- Accession codes, unique identifiers, or web links for publicly available datasets
- A description of any restrictions on data availability
- For clinical datasets or third party data, please ensure that the statement adheres to our policy

Single-cell RNA-seq (scRNA-seq) data is available via GEO (GSE186293). Whole genome sequencing data is available at dbGAP (EGAS00001006035). Original tracings of electrophysiological assessments have been deposited at HKU DataHub (1630281892) and are publicly available as of the date of publication.

Human research participants

Policy information about studies involving human research participants and Sex and Gender in Research.

Reporting on sex and gender	3 males and 3 females were recruited, no sex-based analysis were performed and all findings were not sex-specific.
Population characteristics	Date of surgical repair of tetralogy of Fallot; The need for subsequent pulmonary valve replacement; Arrhythmia; Association
Recruitment	Patients were identified from the cardiac outpatient clinic and invited to participate in the study. Adult healthy volunteers with no known heart disease were recruited as controls.
Ethics oversight	The Institutional Review Board of the University of Hong Kong (UW19-506)

Note that full information on the approval of the study protocol must also be provided in the manuscript.

Field-specific reporting

Please select the one below that is the best fit for your research. If you are not sure, read the appropriate sections before making your selection.

☒ Life sciences ☐ Behavioural & social sciences ☐ Ecological, evolutionary & environmental sciences

For a reference copy of the document with all sections, see nature.com/documents/nr-reporting-summary-flat.pdf

Life sciences study design

All studies must disclose on these points even when the disclosure is negative.

Sample size	2 hiPSC lines for each study group were used to avoid bias interpretation towards any individual differences.
Data exclusions	No
Replication	For scRNA-seq, more than 1 sample for each study group were sequenced for each time points. For electrophysiological studies, at least 3

Replication	batches of experiments were conducted with replication succeeded.
Randomization	Samples were allocated into different study groups based on the 1) cardiac anomalies (tetralogy of Fallot) and 2) the chromosomal anomalies (DiGeorge syndrome/ 22q11.2DS). The chromosomal anomalies were verified with whole-genome sequencing.
Blinding	Blinding is not relevant as it's not a clinical trial study.

Reporting for specific materials, systems and methods

We require information from authors about some types of materials, experimental systems and methods used in many studies. Here, indicate whether each material, system or method listed is relevant to your study. If you are not sure if a list item applies to your research, read the appropriate section before selecting a response.

Materials & experimental systems

n/a	Involved in the study	
☐	☒	Antibodies
☐	☒	Eukaryotic cell lines
☒	☐	Palaeontology and archaeology
☒	☐	Animals and other organisms
☒	☐	Clinical data
☒	☐	Dual use research of concern

Methods

n/a	Involved in the study	
☒	☐	ChIP-seq
☐	☒	Flow cytometry
☒	☐	MRI-based neuroimaging

Antibodies

Antibodies used	Mouse monoclonal anti-OCT3/4 Santa Cruz Biotechnology sc-5279;RRID:AB_628051<br>Goat polyclonal anti-SOX2 Santa Cruz Biotechnology sc-17320;RRID:AB_2286684<br>Mouse monoclonal anti-human SSEA-4 STEMCELL Technologies MC-813-70;RRID:AB_528477<br>Mouse monoclonal anti-TRA-1-81 Cell Signalling Technology 4745S;RRID:AB_2119060<br>Mouse monoclonal anti-α-Fetoprotein (AFP) Sigma-Aldrich A8452;RRID:AB_258392<br>Mouse monoclonal anti-Actin, α-Smooth Muscle Sigma-Aldrich A5228;RRID:AB_262054<br>Mouse monoclonal anti-Tubulin, beta III isoform Sigma-Aldrich MAB1637;RRID:AB_2210524<br>Donkey anti-Goat IgG (H+L) Cross-Adsorbed Secondary Antibody, Alexa Fluor 488 Invitrogen A-11055;RRID:AB_2534102<br>Donkey anti-Mouse IgG (H+L) Highly Cross-Adsorbed Secondary Antibody, Alexa Fluor 488 Invitrogen A-21202;RRID:AB_141607<br>Anti-Cardiac Troponin T antibody [1C11] Abcam ab8295<br>FITC anti-mouse IgG1 Antibody BioLegend 406605
Validation	https://datasheets.scbt.com/sc-5279.pdf<br>http://www.antibodyreview.com/products/559070.0/Sox-2-Antibody-(Y-17)-Santa-Cruz-Biotechnology-sc-17320.html<br>https://www.stemcell.com/anti-human-ssea-4-antibody-clone-mc-813-70.html<br>https://www.cellsignal.com/products/primary-antibodies/tra-1-81-tra-1-81-mouse-mab/4745<br>https://www.sigmaaldrich.com/HK/en/product/sigma/a8452<br>https://www.sigmaaldrich.com/HK/en/product/sigma/a5228?gclid=Cj0KCQjwnNyUBhCZARIsAI9AYlGn42-<br>hZ9XMTBlLYVMcck8zY4qi7SzWzJ3wMRy2WAy8sooLCibXjZYaAjCFEALw_wcB<br>https://www.sigmaaldrich.com/HK/en/product/mm/mab1637?<br>gclid=Cj0KCQjwnNyUBhCZARIsAI9AYlElANPalZ4ZOoV3fnRH_DkR2SJkQgYpJ6m8Huwvff1gMtSnNLujbB8aAhFzEALw_wcB<br>https://www.abcam.com/cardiac-troponin-t-antibody-1c11-ab8295.html

Eukaryotic cell lines

Policy information about cell lines and Sex and Gender in Research

Cell line source(s)	TOF-DG1; hiPSC line reprogrammed from patient TOF-DG1; M<br>TOF-DG2; hiPSC line reprogrammed from patient TOF-DG2; F<br>TOF-ND1; hiPSC line reprogrammed from patient TOF-ND1; M<br>TOF-ND2; hiPSC line reprogrammed from patient TOF-ND2; F<br>Control1; hiPSC line reprogrammed from healthy subject1; M<br>Control2; hiPSC line reprogrammed from healthy subject2; F
Authentication	All the reprogrammed cell lines were validated with iPSC markers as stated in the methods.
Mycoplasma contamination	Negative
Commonly misidentified lines<br>(See ICLAC register)	NA

Flow Cytometry

Plots

Confirm that:

☒ The axis labels state the marker and fluorochrome used (e.g. CD4-FITC).

☒ The axis scales are clearly visible. Include numbers along axes only for bottom left plot of group (a 'group' is an analysis of identical markers).

☒ All plots are contour plots with outliers or pseudocolor plots.

☒ A numerical value for number of cells or percentage (with statistics) is provided.

Methodology

Sample preparation	hiPSC-CMs were dissociated into single cells by 0.025% TrypsinEDTA (Gibco) (TE). Dissociated single cells were subjected to fixation and permeabilization with BD CytoFix/CytopermTM (BD Biosciences). The fixed and permeabilized samples were then incubated with 1% goat serum in PBS-/- at 4oC for 1 hour. The samples were then stained with anti-Cardiac Troponin T antibody (Abcam, ab8295,1:400) in 1% goat serum/PBS-/- overnight at 4 oC. After washing with PBS-/- twice, samples were then stained with FITC conjugated rat anti-mouse IgG1 antibody (BioLegend; 406605,1:50) at 4 oC for 1 hour.
Instrument	FACSCanto™ II (BD Biosciences)
Software	Flowjo
Cell population abundance	Flow-cytometry experiments ended when the number of cTnT positive cells reached 5000.
Gating strategy	Samples incubated with secondary antibody only (without the primary antibody) served as the negative control to determine the baseline fluorescent signal. Any signal stronger than the baseline signal would be regarded as positive signal.

☒ Tick this box to confirm that a figure exemplifying the gating strategy is provided in the Supplementary Information.
